# A mitochondrial proteome profile indicative of type 2 diabetes mellitus in skeletal muscles

**DOI:** 10.1038/s12276-018-0154-6

**Published:** 2018-09-28

**Authors:** Sehyun Chae, Su-Jin Kim, Young Do Koo, Jung Hwa Lee, Hokeun Kim, Byung Yong Ahn, Yong-Chan Ha, Yong-Hak Kim, Mi Gyeong Jang, Kyung-Hoi Koo, Sung Hee Choi, Soo Lim, Young Joo Park, Hak Chul Jang, Daehee Hwang, Sang-Won Lee, Kyong Soo Park

**Affiliations:** 1 Department of New Biology, Daegu Gyeongbuk Institute of Science and Technology, Daegu, 42988 Republic of Korea; 2 Department of Chemistry, Research Institute for Natural Sciences, Korea University, Seoul, 136-701 Republic of Korea; 3 Department of Internal Medicine, Seoul National University College of Medicine, Seoul, 03080 Republic of Korea; 4 Department of Orthopedic Surgery, Seoul National University Bundang Hospital, Seongnam, Gyeonggi-do 13620 Republic of Korea; 5 Department of Microbiology, Catholic University of Daegu School of Medicine, Daegu, 42472 Republic of Korea; 6 Department of Molecular Medicine and Biopharmaceutical Sciences, Graduate School of Convergence Science and Technology, Seoul National University, Seoul, 03080 Republic of Korea; 7 Department of Internal Medicine, Seoul National University Bundang Hospital, Seongnam, 13620 Republic of Korea; 8 Center for Plant Aging Research, Institute for Basic Science, Daegu, 42988 Republic of Korea

## Abstract

The pathogenesis of type 2 diabetes mellitus (T2DM) is closely associated with mitochondrial functions in insulin-responsive tissues. The mitochondrial proteome, compared with the mitochondrial genome, which only contains 37 genes in humans, can provide more comprehensive information for thousands of mitochondrial proteins regarding T2DM-associated mitochondrial functions. However, T2DM-associated protein signatures in insulin-responsive tissues are still unclear. Here, we performed extensive proteome profiling of mitochondria from skeletal muscles in nine T2DM patients and nine nondiabetic controls. A comparison of the mitochondrial proteomes identified 335 differentially expressed proteins (DEPs) between T2DM and nondiabetic samples. Functional and network analyses of the DEPs showed that mitochondrial metabolic processes were downregulated and mitochondria-associated ER membrane (MAM) processes were upregulated. Of the DEPs, we selected two (NDUFS3 and COX2) for downregulated oxidative phosphorylation and three (CALR, SORT, and RAB1A) for upregulated calcium and protein transport as representative mitochondrial and MAM processes, respectively, and then confirmed their differential expression in independent mouse and human samples. Therefore, we propose that these five proteins be used as a potential protein profile that is indicative of the dysregulation of mitochondrial functions in T2DM, representing downregulated oxidative phosphorylation and upregulated MAM functions.

## Type 2 diabetes: Different mitochondrial protein profile in skeletal muscle

Diabetes alters the mitochondrial proteins in insulin-responsive tissues. Sehyun Chae from the Daegu Gyeongbuk Institute of Science and Technology, South Korea, and coworkers characterized the proteins found within the mitochondria of skeletal muscle tissues isolated from nine people with type 2 diabetes and nine non-diabetic controls. They identified 335 proteins that were expressed at significantly different levels in tissues from the two groups. Of these, several involved in energy metabolism were at lower levels in the diabetic cohort, while several involved in communication between the mitochondria and the endoplasmic reticulum, a neighboring celllular organelle, were at higher levels. The researchers confirmed this pattern for five specific proteins in mouse models of diabetes and in human samples. These proteins could form the basis of a diagnostic test for diabetes-associated mitochondrial dysfunction.

## Introduction

A growing volume of evidence has shown that mitochondrial functions can play important roles in the pathogenesis of type 2 diabetes mellitus (T2DM). Mitochondria are intracellular organelles that are responsible for ATP production from glucose and fatty acid oxidation. Thus, they are essential for various cellular events that require ATP, such as cell proliferation or differentiation. T2DM is characterized by both impaired insulin secretion and insulin resistance. Mitochondrial dysfunction in pancreatic beta cells impairs glucose-induced insulin secretion^1^. Moreover, mitochondrial dysfunction in skeletal muscles is also associated with decreased fatty acid oxidation, the accumulation of intracellular lipid metabolites, and inhibition of the insulin signaling pathway^2^. These data suggest close associations of mitochondrial functions with the pathophysiology of T2DM in both insulin-secreting and responsive tissues.

In many diseases, genomic or proteomic profiles provide molecular signatures to define the states related to the diseases. The human mitochondrial genome (mtDNA) has provided a molecular basis for understanding various diseases, such as neurodegenerative diseases, cancers, and T2DM^3–5^. Over 150 mtDNA mutations have been associated with such diseases, as well as other maternally inherited syndromes^6^. Nonetheless, considering that the mtDNA includes 37 genes and encodes only 13 proteins, the capacity for mtDNA mutations to account for the pathophysiology of these diseases is limited. By contrast, proteomic profiles also provide unique, complementary information that links genotypes to phenotypes. Thousands of both nucleus-encoded and mitochondria-encoded proteins are localized in mitochondria, defining mitochondrial functions associated with the diseases. Thus, compared with mitochondrial genome, the mitochondrial proteome can provide more comprehensive information for thousands of mitochondrial proteins, which can be used to evaluate mitochondrial functions associated with T2DM.

Recent advances in mass spectrometry (MS)-based proteomic methods have improved the size of detected mitochondrial proteomes to more than 1500 mitochondrial proteins^7^. The large proteome size suggests that not only metabolic pathways but also other cellular pathways, such as those for calcium homeostasis, apoptosis, and signal transduction, are active in mitochondria. Thus, comprehensive mitochondrial proteomes in insulin-responsive tissues can provide a molecular basis for understanding T2DM-associated mitochondrial dysregulation of such metabolic and cellular pathways. To search for proteome profiles associated with the pathogenesis of T2DM, several studies have described the global proteome profiles of tissue and serum samples from T2DM patients or mouse models^8–12^. For example, Li et al.^8^ have identified 68 proteins that are upregulated in the sera of T2DM patients, found a significant association of the complement system with T2DM, and then selected ficolin-3, an upstream activator of the complement cascade, as a protein that is indicative of T2DM-associated complement activation. However, to our knowledge, no comparative study of mitochondrial proteomes in insulin-responsive tissues from T2DM patients and nondiabetic controls has been performed to identify the proteome profiles indicative of mitochondrial functions in T2DM.

Here we performed extensive proteome profiling of mitochondria isolated from skeletal muscles in T2DM patients (*n* = 9) and nondiabetic controls (*n* = 9) to understand the mitochondrial proteome profiles associated with T2DM. For protein digestion, we employed filter-aided sample preparation (FASP)^13^ to improve detection of the proteins incorporated in both inner and outer mitochondrial membranes. Furthermore, the use of ultra-high pressure nano-LC-MS/MS coupled with extensive fractionation provided comprehensive mitochondrial proteomes, including 23,122 peptides for 1150 proteins, thereby enabling a thorough comparative analysis of mitochondrial proteomes between skeletal muscles from T2DM patients and nondiabetic controls. From the measured mitochondrial proteomes, we selected hundreds of differentially expressed proteins (DEPs) in skeletal muscles from T2DM patients. Through functional and network analyses, among the DEPs, we selected a protein profile indicative of mitochondrial functions in diabetic skeletal muscles.

## Materials and methods

### Sample collection

We collected two independent sets of skeletal muscles from T2DM and control subjects who underwent elective orthopedic surgery in Seoul National University Bundang Hospital. The first set of nine T2DM patients and nine nondiabetic control subjects were used for profiling mitochondrial proteomes. We enrolled T2DM patients who were diagnosed with diabetes based on American Diabetes Association (ADA) criteria^14^. The control group was enrolled based on the same ADA criteria. To confirm the validity of the selected mitochondrial proteins indicative of T2DM by western blotting, we collected the second set, which included 14 T2DM patients and 12 nondiabetic control subjects. Informed consent was obtained from all subjects. This study was conducted according to the Declaration of Helsinki and was approved by the ethics committees of SNUH [SNUBH IRB# B-0710/050-009]. The clinical characteristics of all enrolled patients for proteome profiling and for confirming candidate proteins are described in Supplementary Table 1.

### Animal experiments

C57BL/6 mice (Orient Bio, Seongnam, Gyeonggi-do, Korea) were fed a normal chow diet [Purina LabDiet, Purina Mills (St Louis, MO)] and a HFD [60% high fat and high sucrose, Research diet (New Brunswick, NJ)] for 12 weeks at 6 weeks after birth. All animal studies were approved by the Institutional Animal Care and Use Committee of Seoul National University Hospital (Permit Number: SNU-150126-1-3).

### Isolation of mitochondria

We isolated mitochondria from wild-type cybrid cells^15^ and skeletal muscle tissues as previously described^16^. The cells and tissues were homogenized in mitochondria isolation buffer (250 mM sucrose and 1 mM EDTA in 25 mM Tris-HCl pH 7.4) at 4 °C. Subsequently, the lysates were centrifuged at 1000 × *g* to the separate nuclear and cytosolic fraction, and the cytosolic fraction was further centrifuged at 10,000 × *g* at 4 °C to obtain a crude mitochondrial pellet. The isolated mitochondria were further purified through a 10–40% Percoll gradient by centrifugation at 20,000 rpm in a Beckman SW41 rotor for 120 min. The upper and lower fractions (P1 and P3 fractions, respectively) of the mitochondrial layer in the gradient were carefully discarded. The remaining mitochondrial layer (P2 fraction) was washed twice and stored at –70 °C until use.

#### Mitochondrial protein and peptide preparation

Isolated mitochondria from skeletal muscle tissues and cybrid cells were dissolved in 4% SDS lysis buffer in Tris HCl at pH 7.6, and the solution was transferred into 1.5-ml e-tubes containing 2-mm glass beads. The mitochondria were disrupted by repeated strokes at 1000 rpm for 30 s using a Mini-BeadBeater^TM^ Mill (Cole-Parmer Instrument, Vernon Hills, IL) and cooled on ice for 1 min^17^. This procedure was repeated until no pellets were visible (~10–15 times). The supernatants were transferred to a new tube and centrifuged at 5000 rpm for 10 min at 20 °C to remove the debris. The resulting supernatant was transferred to a new tube, sonicated using a Q55 Sonicator (Qsonica, Newtown, CT) for 30 s, and subjected to cooling for 1 min. The sonicated lysis procedure was repeated 10 times on ice. The lysate was centrifuged at 14,000 × *g* for 10 min at 20 °C, the supernatant was transferred to a new tube, and the protein concentration was determined using a BCA protein assay (Pierce, Rockford, IL) and bovine serum albumin.

Mitochondrial peptide samples were prepared using a modified FASP method^13^. Briefly, 100 μg of protein lysate was reduced in 100 μL of SDT (4% SDS and 0.1 M DTT in 0.1 M Tris-HCl, pH 7.6) solution for 45 min at 37 °C and then boiled for 10 min to increase the denaturation. After sonication for 10 min, the sample was centrifuged for 5 min at 16,000 × g. The reduced protein sample was mixed with 200 μL of 8 M urea in a Microcon filter YM-30 (Millipore Corporation, Bedford, MA), and the filter was centrifuged at 14,000 × *g* for 60 min. All centrifugation steps were performed at 20 °C. To remove residual SDS, 200 μL of 8 M urea in 0.1 M Tris-HCl (pH 8.5) was added to the filter before the filter was centrifuged at 14,000×*g*. This washing step was repeated twice. Subsequently, 100 μL of 50 mM iodoacetamide in 8 M urea in 0.1 M Tris-HCl was added to the concentrate for alkylation at 25 °C for 25 min in the dark, followed by centrifugation at 14,000 × g for 30 min to remove the alkylation reagent. The resulting sample was washed twice with 200 μL of 8 M urea in 0.1 M Tris-HCl and then twice with 100 μL of 50 mM NH_4_HCO_3_. The alkylated protein sample was subjected to proteolytic digestion using trypsin (1:50 enzyme-to-protein ratio (w/w), Promega, Madison, WI) at 600 rpm for 1 min at 37 °C, followed by an overnight incubation for 12 h with no shaking in a thermo-mixer comfort (Eppendorf, Hamburg, Germany). The second trypsin digestion (1:100 enzyme-to-protein ratio) was carried out for 6 h. The digested peptides were eluted by centrifugation at 14,000 × *g* for 20 min. The filter was additionally rinsed with 60 μL of 50 mM NH_4_HCO_3_ and centrifuged at 14,000 × *g* for 30 min. The eluent was mixed with the first eluent. The peptide sample was dried in a vacuum concentrator SPD1010 (Thermo, San Jose, CA) and stored at −80 °C.

### Fractionation of mitochondrial peptides by offgel electrophoresis

The 500 μg of tryptic peptides from the P2 mitochondrial fraction of the cybrid cell was fractionated using a 3100 OFFGEL Fractionator (Agilent Technologies, Santa Clara, CA) according to the manufacturer’s protocol. A 24-well tray was used with a 24-cm IPG gel strip of a linear pH gradient ranging from 3 to 10 (Immobiline DryStrip, GE Healthcare Life Sciences, Uppsala, Sweden), and the gel strip was rehydrated for 15 min with 25 μL of the rehydration solution per well. The tryptic peptide was dissolved in 0.72 mL deionized water and mixed with 2.88 mL of the offgel stock solution. In each well, 150 μL of the resultant solution was placed. Focusing was performed at 20 °C with a voltage range from 500 to 8000 V and maximum current of 50 μA for 50 kVh. After focusing, the peptide fractions from the 24 wells, as well as extra acid (anodic ends) and extra base (cathodic ends) fractions, were collected. To remove glycerol, spin desalting columns were used. The peptides were then dried using the vacuum concentrator.

### LC-MS/MS experiments

Mitochondrial peptides were separated using a modified version of the nanoACQUITY UPLC (NanoA, Waters, Milford, MA) system^18^. An analytical column (75 μm ID × 360 μm OD × 70 cm length) was manufactured in-house by packing a fused-silica capillary (Polymicro Technologies, Phoenix, AZ) with C18 materials (3 μm diameter, 300 Å pore size, Jupiter, Phenomenex, Torrance, CA) using acetonitrile slurry. The solid phase extraction (SPE) column was prepared by packing the same C18 materials into the 1-cm-long liner column (250 μm ID) of an internal reducer (1/16” to 1/32”, VICI, Houston, TX). The column temperature was set to 50 °C using a semi-rigid gasline heater (1/4” ID, 60 cm long, WATLOW, Winona, MN)^19^. The LC separation gradient was 98% solvent A (0.1% formic acid in H_2_O) for 5 min, 2 to 50% solvent B (0.1% formic acid in 99.9% acetonitrile) for 115 min, 50 to 80% solvent B in 10 min, and 80% solvent B for 10 min. The flow rate of the mobile phase was set to 400 nL/min. A 7-tesla Fourier transform ion cyclotron resonance mass spectrometer (FTICR, LTQ-FT, Thermo Electron, San Jose, CA) was used to collect the mass spectra. The eluted peptides from the LC were ionized at an electrospray potential of 2.0 kV. The electrospray ionization (ESI) emitter was made by chemical etching of a fused-silica capillary emitter (20 μm ID × 150 μm OD)^20^. The temperature of the desolvation capillary was set to 250 °C. MS precursor ion scans (*m/z* 500–2000) were acquired in full-profile mode with an AGC target value of 1 × 10^6^, a mass resolution of 1 × 10^5^, and a maximum ion accumulation time of 500 ms. The mass spectrometer was operated in a data-dependent tandem MS mode, and the seven most abundant ions detected in a precursor MS scan were dynamically selected for MS/MS experiments incorporating a dynamic exclusion option (exclusion mass width low = 1.10 Th; exclusion mass width high = 2.10 Th; exclusion list size = 120; and exclusion duration = 30 s) to prevent reacquisition of MS/MS spectra of the same peptides. Collision-induced dissociations of the precursor ions were performed in an ion trap (LTQ) with the collisional energy and isolation width set to 35% and 3 Th, respectively. The Xcalibur software package (v. 2.0 SR2, Thermo Electron) was used to construct the experimental methods. All LC-MS/MS data were deposited in the ProteomeXchange Consortium via the PRIDE^21^ partner repository with the dataset identifier PXD004312 and 10.6019/PXD004312 (Username: reviewer16736@ebi.ac.uk, Password: F64K10U7).

### Peptide identification

For peptide identification, the integrated post experiment monoisotopic mass refinement (*i*PE-MMR) method was first used to process the LC-MS/MS data, as previously described^22^, before the subsequent protein database search (Supplementary Figure S1a). Briefly, DeconMSn^23^ was used to generate MS/MS data (1st DTA files), and precursor masses in the DTA files were further corrected and refined through PE -MMR^24^. The mass-refined DTA files (2nd DTA files) were subjected to multivariate mass error correction using DtaRefinery^25,26^. The DTA files were then converted to mgf files using an in-house software, which was used for the MS-GF+ (ver. 9881)^27^ search against a composite target-decoy database that contains forward and reverse protein sequences of the uniprot human reference database (April, 2014; 88,608 entries) and 179 common contaminants. A MS-GF+ search was performed with the following parameters: precursor mass tolerance of 10 ppm, semi-tryptic, static modification of carbamidomethylation (57.021460 Da) to cysteine, and variable modifications of oxidation (15.994920 Da) to methionine and carbamylation (43.005810 Da) to N-termini. The peptide spectrum matches (PSMs) were obtained using the false discovery rate (FDR) of 1%.

### Construction of a master accurate mass and time tag (AMT) database (DB)

**Fig. 1:**
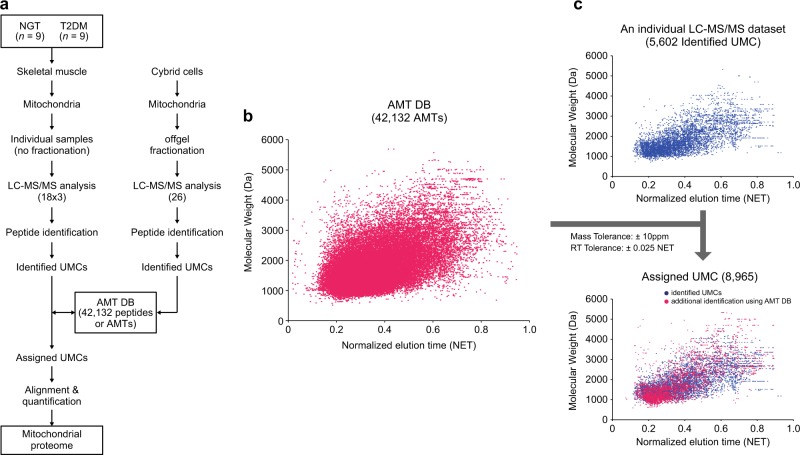
Master AMT DB of the mitochondrial proteome. **a** The overall scheme of AMT DB construction. A total of 80 datasets (18 × 3 + 24) were generated from LC-MS/MS analysis. Each dataset was processed by *i*PE-MMR, after which a target-decoy MS-GF+ search was performed (peptide identification). The resultant peptides (or identified UMCs) were used to construct the AMT DB. **b**, **c** Utilization of the AMT DB to assign peptide IDs to unidentified UMCs. The 42,132 AMTs (magenta dots) in the AMT DB are visualized in a 2D (NET and molecular weight) scatter plot (left). For a LC-MS/MS dataset, the identified UMCs (blue dots) are shown in the upper right scatter plot. By matching the unidentified UMCs in this dataset with AMTs using the indicated mass and NET tolerances, a subset of unidentified UMCs was assigned to peptide IDs of matching AMTs. These matched UMCs (red dots) are shown in the bottom right scatter plot

We next constructed a master AMT DB for the mitochondrial proteome using the identified peptides as described previously (Supplementary Figure S1b)^28^. Briefly, the MS features of a peptide that emerged over a period of LC elution time in a LC-MS/MS experiment were first grouped into a unique mass class (UMC) by PE-MMR analysis (Supplementary Figure S1a)^24^. Ideally, a peptide is represented by a UMC. The MS features of each UMC included all of the mass spectral features of a peptide, such as the charge state, abundance (intensity), scan number, and measured monoisotopic mass. Precursor masses of MS/MS spectra were matched with UMC masses and replaced with the matched UMC masses (2nd DTA files, Supplementary Figure S1a). In this process, DTA information was linked to the matched UMC. After MS-GF+ search and target-decoy analysis, the DTA file linked to the UMC resulted in a peptide sequence (FDR ≤ 1%), and the peptide ID was recorded in the UMC, called identified UMCs (UMC assignment, Supplementary Figure S1b). Next, normalized elution times (NETs) were calculated and calibrated for the identified UMCs, as previously described^29^, and the calibrated NETs were recorded in the UMCs (NET calibrated UMCs, Supplementary Figure S1b). The information for these NET calibrated UMCs from all 80 LC-MS/MS datasets (triplicate LC-MS/MS experiments of 18 samples and 26 LC-MS/MS experiments of offgel fractionations) was compiled into the master AMT DB (Oracle Database 10 g Enterprise Edition Release 10.2.0.1.0; Fig. 1a). When a peptide was measured multiple times, the average mass and median NET were recorded for the AMT for the peptide (Supplementary Figure S1b).

### Peptide assignment to unidentified UMCs using the AMT DB

Due to the undersampling of the current proteomic analysis platform, a large portion of UMCs from an LC-MS/MS experiment was not identified by MS/MS experiments and by protein database searches. For example, for an LC-MS/MS dataset, 54,077 UMCs were found, and only 5602 of them (ca. 10.4%) were identified by protein database search (identified UMCs; Fig. 1b, right top). Peptide IDs were assigned to these unidentified UMCs using the AMT DB. Each unidentified UMC was matched to the identified UMCs in the AMT DB using the mass and NET tolerances (±10 ppm and ±0.025 NET), and the peptide ID of the matched UMC in the AMT DB was assigned to the unidentified UMC (Fig. 1b, right bottom; Assigned UMCs, Supplementary Figure S1c). This process resulted in 3363 additional UMCs identified by the master AMT DB information, thereby increasing the total number of UMCs with peptide ID assignments to 8965 (assigned UMCs).

### Alignment of the identified peptides across samples

An MS intensity-based label-free quantitation method was applied to an LC-MS/MS dataset as previously described^28^. Briefly, for each assigned UMC, the intensity (UMC intensity) was calculated during PE-MMR analysis as the summation of abundances for all mass spectral components of the UMC to represent the abundance of the corresponding peptide^24^. The assigned UMCs from the 54 LC-MS/MS datasets (i.e., triplicate LC-MS/MS datasets of 18 individual mitochondria peptide samples) were then combined into an alignment table in which each row contains the peptide IDs with their corresponding UMC intensities from each LC-MS/MS experiment (UMC alignment, Supplementary Figure S1c). Quantile normalization of the aligned data was performed to correct systematic variations in peptide abundances across individual LC-MS/MS datasets as previously described^30^.

### Analysis of mitochondrial proteomes

To identify DEPs between T2DM patients and nondiabetic controls, we first selected a set of peptides (1) with corresponding proteins having more than two non-redundant peptides and (2) with abundances that were detected in more than 50% of all subjects in each group to ensure their statistical power. For these selected peptides, we then performed a statistical hypothesis test that integrates Student’s *t*-test and log_2_-median ratio test, as previously described^15^. Briefly, for the selected peptides, we (1) applied Student’s *t*-test and log_2_-median ratio test to compute *T*-values and log_2_-median ratios, (2) calculated the adjusted *P*-values of the *T*-value and log_2_-median ratio for each peptide using empirical null distributions for the two tests estimated from random permutation experiments as previously described^31^, (3) combined the adjusted *P*-values from the two tests using Stouffer’s method^32^ for each peptide, (4) selected differentially expressed peptides with FDR ≤ 0.1 and ratio ≥ 1.5 after computing FDRs for the combined *P*-values using Storey’s method^33^, and then identified DEPs containing the differentially expressed peptides. Moreover, to understand the cellular processes associated with DEPs, functional enrichment analysis was performed using DAVID software to identify GO biological processes (GOBPs) represented by the DEPs^34^. The GOBPs enriched by the DEPs were identified as those with a *P* ≤ 0.05.

### Reconstruction of the network model

To reconstruct a network model for DEPs, we first selected a subset of DEPs annotated with T2DM-related GOBPs enriched by the DEPs and then collected their protein–protein interactions for the selected DEPs from four protein–protein interactome databases: BIND (Biomolecular Interaction Network Database)^35^, HPRD (Human Protein Reference Database)^36^, BioGRID (Biological General Repository for Interaction Datasets)^37^, and MINT (Molecular INTeraction Database)^38^. The initial network model was built with the selected DEPs and their interactors using Cytoscape^39^. We then arranged the nodes according to their associated GOBPs and pathways such that the nodes with similar functions were located closely. The groups of nodes involved in the same GOBPs were labeled with the corresponding GOBP.

### Western blot analysis

For this analysis, we prepared total lysate and crude mitochondrial samples from mouse skeletal muscle tissues and human skeletal muscles tissues. To prepare the total lysate, tissues were lysed in 20 mM Tris-HCl buffer (pH 7.4) containing 10 mM Na_4_P_2_O_7_, 100 mM NaF, 2 mM Na_3_VO_4_, 1% NP-40, and a protease inhibitor cocktail (10 µg/µl aprotinin, 10 µg/µl leupeptin and 1 mM PMSF). The lysates were sonicated two times for 15 s each, and the cell debris was removed by centrifugation (13,000 rpm) for 30 min at 4 °C. To prepare crude mitochondria, the tissues were homogenized in mitochondria isolation buffer (250 mM sucrose, 25 mM Tris-HCl pH 7.4, 1 mM EDTA) at 4 °C. Subsequently, the lysates were centrifuged at 1000 × *g* to separate the nuclear and cytosolic fraction, and the cytosolic fraction was further centrifuged at 10,000 × *g* at 4 °C to obtain a crude mitochondria pellet. The pellet was dissolved in protein lysis buffer. Approximately 20–30 µg of the total lysate and crude mitochondrial fractions were separated by SDS-PAGE. Separated proteins were transferred onto the nitrocellulose membrane (Whatman, Dassel, Germany). The membrane was blocked with 5% skim milk in Tris-buffered saline-Tween20 (TBS-T) for 1–2 h at room temperature and then was probed overnight at 4 °C with specific antibodies against the selected mitochondrial proteins (Supplementary Table S2).

## Results

### Comprehensive profiling of the mitochondrial proteome

To investigate mitochondrial proteomes associated with T2DM, we first selected nine T2DM patients (*n* = 9; four males and five females) and nine nondiabetic controls (*n* = 9; five males and four females). The characteristics of the T2DM patients and nondiabetic controls, such as age, gender, body mass index, fasting blood glucose, and other laboratory measures, are summarized in Supplementary Table S1. From each of the T2DM patients and normal controls, we obtained skeletal muscle tissues and then isolated mitochondria using a previously described method^16^ (see Materials and methods). To facilitate detection of membranous proteins that are closely associated with mitochondrial physiology, we used the FASP^13,40^ method for protein digestion (see Materials and methods). We then performed triplicate LC-MS/MS experiments on each of the resulting peptide samples from nine T2DM patients and nine nondiabetic controls, resulting in 54 (18 × 3) LC-MS/MS datasets (Fig. 1a, left branch; Supplementary Figures S2 and S3). In addition to skeletal muscle mitochondria, we further isolated mitochondria from wild-type cybrid cells as previously reported^15^, used the FASP method for protein digestion, carried out offgel fractionation of the resulting peptide samples into 26 fractions, and then performed LC-MS/MS experiments for the fractions, resulting in 26 LC-MS/MS datasets (Fig. 1a, right branch; Supplementary Figure S4).

Next, we constructed a master AMT DB for the mitochondrial proteome using the total 80 LC-MS/MS datasets from skeletal muscle (54 datasets) and cybrid mitochondria (26 datasets) as previously described^41^. To achieve this goal, we generated UMCs of peptide MS features using *i*PE-MMR analysis^22^, assigned peptide IDs and NETs to the UMCs (identified UMCs), which were obtained from the target-decoy MS-GF+ search (FDR < 0.01) and NET calculation, respectively, and then complied all the identified UMCs from the 80 datasets into an AMT DB (Fig. 1a, bottom; see Materials and methods; Supplementary Figure S1). This procedure resulted in the master AMT DB comprising 42,132 peptides (or AMTs) (Fig. 1b, dots). For each dataset (Fig. 1c, top), we then used the master AMT DB to assign peptide IDs to unidentified UMCs (red dots in Fig. 1c, bottom) with a mass tolerance of 10 ppm and NET tolerance of 0.025. As a result, 23,122 peptides were identified from 54 LC-MS/MS datasets that were generated from nine T2DM and nine control skeletal muscle samples. Among them, we then selected 7635 peptides that were detected in more than half of the T2DM (*n* ≥ 5) and control (*n* ≥ 5) samples to focus on the peptides with statistical power in the comparison of their abundances between the T2DM and control (Supplementary Figure S5). These 7635 peptides were mapped to 1671 proteins. Of them, as mitochondrial proteins with high confidence, we finally selected 1171 proteins (1150 protein-coding genes) with two or more sibling peptides (Supplementary Table 2).

### Characteristics of mitochondrial proteome from skeletal muscle

**Fig. 2:**
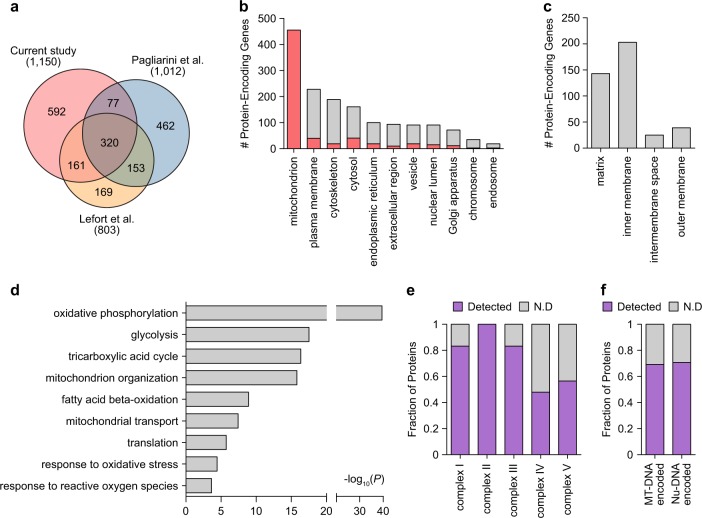
Comprehensive profiles of the mitochondrial proteome. **a** Comparison of our proteome with two previous mitochondrial proteomes. For the proteins detected in individual studies, the numbers of genes encoding the proteins are shown. Venn diagram showing the relationships of our mitochondrial proteome with the largest previous mitochondrial proteomes. **b** Relative proportions of cellular components (GOCCs) in which the measured mitochondrial proteins are mainly localized. In each stacked bar, the red bar represents the proportion of proteins that can be localized to mitochondria according to GOCCs (e.g., 40 of 228 plasma membranous proteins can be co-localized to mitochondria). **c** Distribution of the mitochondrial proteins in sub-mitochondrial localization. **d** Cellular processes (GOBPs) in which the identified proteins are mainly involved. The bars represent–log_10_(*P*), where *P* represents the significance of each GOBP enriched by the mitochondrial proteins. The *P*-value was computed using DAVID software. **e**, **f** Coverage of the mitochondrial proteome in oxidative phosphorylation complexes. In the stacked bars, the purple bars represent the proportions of proteins detected for all of the proteins included in complex I-V (**e**) and encoded by the nucleus (Nu-DNA) or mitochondria genome (MT-DNA) (**f**)

To assess the comprehensiveness of our mitochondrial proteome from skeletal muscle tissues, we compared our 1150 proteins with the most comprehensive mouse and human mitochondrial proteomes reported in two previous studies^42,43^. Paglianini et al.^42^ provided a mitochondrial compendium of 1098 protein-coding genes from an integrated analysis of (1) LC-MS/MS profiles of mitochondria isolated from 14 mouse tissues, (2) mitochondrial proteins reported in previous literature, and (3) GFP tagging microscopy data. The 1098 genes were mapped to 1012 human orthologs based on the human-mouse ortholog information in the Mouse Genome Informatics database. Lefort et al.^43^ profiled the proteome of mitochondria isolated from human skeletal muscles and then identified 823 proteins, which were mapped to 803 protein-coding genes. The comparison showed that our 1150 mitochondrial protein-coding genes included 397 (39.2% of 1012) and 481 (60.0% of 803) protein-coding genes (in total 558) identified from 14 mouse tissues and human skeletal muscle tissues, respectively (Fig. 2a). The remaining 592 of 1150 protein-coding genes were not reported in the two previous mitochondrial proteomes. These data suggest that our proteome from skeletal muscle tissues can serve as a comprehensive source of human tissue mitochondrial proteomes.

To understand the characteristics of our mitochondrial proteome, we then examined the cellular localization of the 1150 proteins based on the Gene Ontology Cellular Components (GOCCs)^44^. The largest number of proteins (455 of 1150; 39.6%) was found to be localized in mitochondria, followed by plasma membrane and cytosol/cytoskeleton (Fig. 2b; Supplementary Table S3A). Comparison of mitochondrial proteomes detected by LC-MS/MS showed that the fraction (39.6%) of proteins annotated with mitochondrion based on GOCCs was comparable to those reported in Paglianini et al.^42^ and Lefort et al.^43^ mentioned above (see 'Discussion'). Moreover, the localization distribution of the mitochondrial 455 proteins in our study further revealed that our proteome included the proteins localized in the whole spectrum of sub-mitochondrial compartments, ranging from the mitochondrial outer and inner membranes to the inner membrane space and mitochondrial matrix (Fig. 2c). Next, we examined the cellular processes represented by the 1150 proteins by performing enrichment analysis of Gene Ontology Biological Processes (GOBPs) using DAVID software^34^. The cellular processes significantly (*P* < 0.05) represented by the mitochondrial proteome included processes related to T2DM-associated mitochondrial functions in skeletal muscles, such as oxidative phosphorylation (OXPHOS), glycolysis, the tricarboxylic acid (TCA) cycle, mitochondrion organization/transport, fatty acid beta-oxidation, response to oxidative stress, and translation (Fig. 2d; Supplementary Table S3B). For the OXPHOS complexes, our proteome provided comprehensive coverage (48–100%) of the proteomes in all five complexes (Fig. 2e), regardless of the mitochondria-encoded or nuclear-encoded OXPHOS proteins (Fig. 2f). These data suggest that in addition to the comprehensiveness, our mitochondrial proteome provided a wide range of the mitochondrial proteome involved in various pathophysiological processes that occur in sub-mitochondrial compartments.

### The mitochondrial proteome of skeletal muscle that is altered in T2DM

**Fig. 3:**
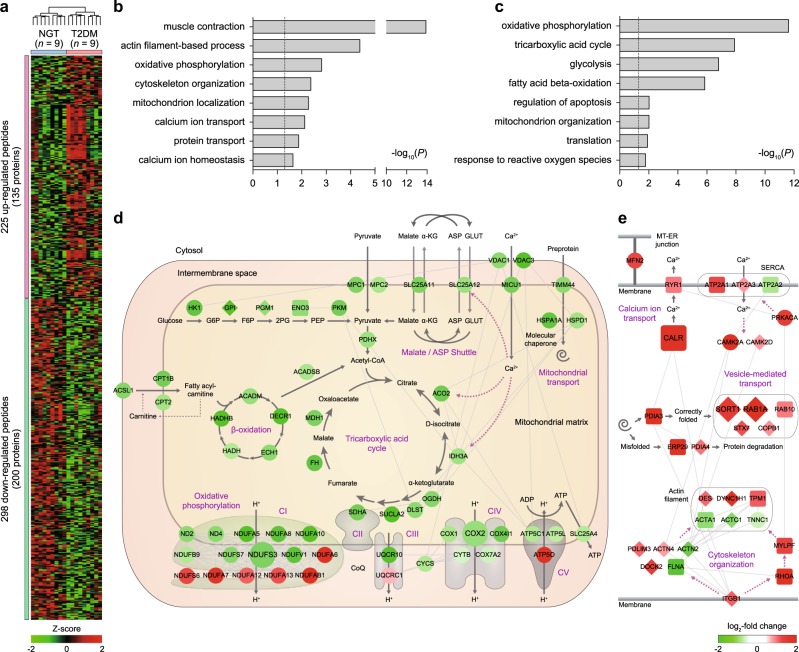
Network models delineating cellular processes represented by T2D-associated mitochondrial proteins (DEPs). **a** Heat maps showing differential expression of 523 peptides with altered abundances between T2DM patients and nondiabetic controls. Colors represent upregulation (red) and downregulation (green) in T2DM compared with nondiabetic controls. The color bar denotes the gradient of the auto-scaled intensities with a mean = 0 and standard deviation = 1. **b**, **c** Cellular processes (GOBPs) represented by upregulated (**b**) and downregulated (**c**) proteins. The bars represent –log_10_(*P*), where *P* represents the significance of each GOBP enriched by the DEPs. **d**, **e** Network models describing the interactions of the DEPs in mitochondria (**d**) and MAM (**e**) and their first neighbors involved in the selected GOBPs. The node colors represent upregulation (red) and downregulation (green) in T2DM compared with nondiabetic controls. The color bar denotes the gradient of the log_2_-fold changes between T2DM and nondiabetic controls. The edges represent protein–protein interactions (gray) collected from four interactome databases

In skeletal muscles, T2DM has been associated with mitochondrial dysfunction involving alterations in the abundance of mitochondrial proteomes^45–47^. To identify the mitochondrial proteins with altered abundances in T2DM, in comparison to nondiabetic controls, we first aligned the peptides measured in the 54 LC-MS/MS datasets (27 for nine T2DM patients and 27 for nine nondiabetic controls) and then identified 523 differentially expressed peptides between T2DM and nondiabetic controls using a previously reported method^28^ (see Materials and methods). The 523 peptides were mapped into 335 DEPs between T2DM and nondiabetic controls. Among the 335 DEPs, 135 proteins were upregulated in T2DM compared with the nondiabetic controls, whereas 200 proteins were downregulated (Fig. 3a; Supplementary Table S4). To understand the functional association of the DEPs with T2DM, we identified cellular processes represented by the upregulated and downregulated proteins using DAVID software (see Materials and methods). This analysis revealed that the 135 upregulated proteins were mainly involved in processes related to molecular transport (calcium ion transport, protein transport, and calcium ion homeostasis) and cytoskeletal organization (muscle contraction, actin filament-based process, and actin cytoskeleton organization) (Fig. 3b; Supplementary Table S5A), consistent with previous findings that molecular transport and cytoskeletal organization have been linked to T2DM pathogenesis^47^. By contrast, the 200 downregulated proteins were mainly involved in metabolic processes, such as glucose (glycolysis) and fatty acid metabolic processes (fatty acid beta-oxidation) and the TCA cycle, which can greatly contribute to T2DM pathogenesis (Fig. 3c; Supplementary Table S5B). Interestingly, however, both the upregulated and downregulated proteins (6 and 16 proteins, respectively) significantly represented OXPHOS. The conflicting alteration patterns of OXPHOS proteins were consistent with those found for OXPHOS genes in previous studies^45^.

To explore the collective function of the aforementioned T2DM-associated processes, we reconstructed network models (Fig. 3d, e) describing interactions among the DEPs involved in the GOBPs related to molecular transport, metabolism, and cytoskeletal organization (see Materials and methods). First, the network model in Fig. 3d showed a downregulation of metabolic pathways, including the glycolytic pathway (HK1, GPI, PGM1, ENO3, and PKM), TCA cycle (ACO2, IDH3A, OGDH, DLST, SUCLA2, SDHA, FH, and MDH1), and fatty acid β-oxidation (ACADM, DECR1, ECH1, HADH, and HADHB) in T2DM compared with the nondiabetic controls. Consistent with the downregulation of these pathways, the network model further showed that the transport of glucose and aspartate (SLC25A12), pyruvate (MCP1/2), malate and α-ketoglutarate (SLC25A11), and fatty acids (ACSL1 and CPT1B/2) were also downregulated in T2DM. Additionally, the proteins in the five OXPHOS complexes were largely downregulated in T2DM, while conversely, several proteins (ATP5O and NDUFA6/A7/A12/AB1/S6) were upregulated, suggesting complex alteration patterns of the OXPHOS system in T2DM. Moreover, mitochondrial protein transport (TIMM44) was downregulated in T2DM, together with molecular chaperones (HSPA1A/D1). Furthermore, the network model also revealed a downregulation of calcium (MICU1 and VDAC1/3) and ATP transport (SLC25A4 and VDAC1/3). Collectively, the downregulation of these key mitochondrial metabolic processes in the network model suggests a decreased overall activity of mitochondrial metabolism in T2DM compared with nondiabetic controls.

By contrast, the network model in Fig. 3e showed an upregulation of several processes that were originally reported to be associated with endoplasmic reticulum (ER): ER calcium trafficking (RYR1 and ATP2A1/A3) (Fig. 3e, top), ER unfolded protein response (PDIA3/4 and ERP29), and vesicle-mediated protein trafficking (SORT1, RAB1A/10, STX7, and COPB1) (Fig. 3e, middle). These ER cellular processes represented by the upregulated mitochondrial proteins in T2DM suggest a potential association of ER with mitochondrial functions in T2DM. Recently, it has been reported that a subset of mitochondria are in close contact with the ER, forming the interface called the mitochondria-associated ER membrane (MAM)^48–50^, and the functions of the MAM are increased when mitochondrial functions are decreased under pathological conditions^51^. Interestingly, among MAM proteins that have been previously reported in several studies, a significant (40.1%; 15 of 37 previous MAM proteins) portion of them were detected in our mitochondrial proteome (Supplementary Table S6). These data suggest that our mitochondrial samples that included MAMs and upregulated MAM proteins (Fig. 3e) can also be used to establish a mitochondrial profile that is indicative of T2DM-associated mitochondrial functions (see 'Discussion'). Finally, the network model (Fig. 3e, bottom) showed upregulation of a cytoskeletal organization pathway (ITGB1, RHOA, MYLPF, DOCK2, PDLIM3, ACTN4, DES, DYNC1H1, and TPM1) in T2DM compared with nondiabetic controls, while conversely, several molecules (FLNA, ACTA1/C1, ACTN2, and TNNC1) in the pathway were downregulated.

### A mitochondrial proteome profile indicative of T2DM pathophysiology

The above-described network models revealed the following representative T2DM-associated cellular pathways: mitochondrial metabolic pathways (Fig. 3d) and MAM-associated cellular pathways for ER calcium trafficking (Fig. 3e, top) and ER unfolded protein response and transport (Fig. 3e, middle). Thus, we selected a mitochondrial proteome profile indicative of T2DM pathophysiology as representative proteins of these three T2DM-associated cellular processes (Supplementary Figure S6). First, in skeletal muscles of human subjects and rodents with T2DM, mitochondrial metabolic dysregulation, involving impairment of OXPHOS, has been observed in insulin-resistant states^2,52,53^, consistent with the downregulated OXPHOS in the network model (Fig. 3d). As a mitochondrial proteome profile indicative of T2DM-associated mitochondrial functions, we thus selected the two representative proteins for the downregulated OXPHOS (Supplementary Figure S6): (1–2) downregulated NDUFS3 (Complex I) and COX2 (Complex IV). Second, mitochondrial dysfunction has been shown to be linked to impaired calcium transport^54–56^, which can subsequently alter calcium homeostasis in the MAM^57^, consistent with the upregulated calcium transport at the MAM in the network model (Fig. 3e, top). Third, mitochondrial dysfunction has also been reported to result in decreased protein transport into mitochondria^58^, which can then alter protein transport in the MAM, consistent with the upregulated protein transport at the MAM in the network model (Fig. 3e, middle). Thus, we selected the following three representative proteins for calcium and protein transports as an additional mitochondrial proteome profile indicative of T2DM-associated mitochondrial functions (Supplementary Figure S6): (3) upregulated CALR for calcium transport and (4–5) upregulated SORT1 and RAB1A for protein transport.

### Validation of the selected mitochondrial protein profile indicative of T2DM

**Fig. 4:**
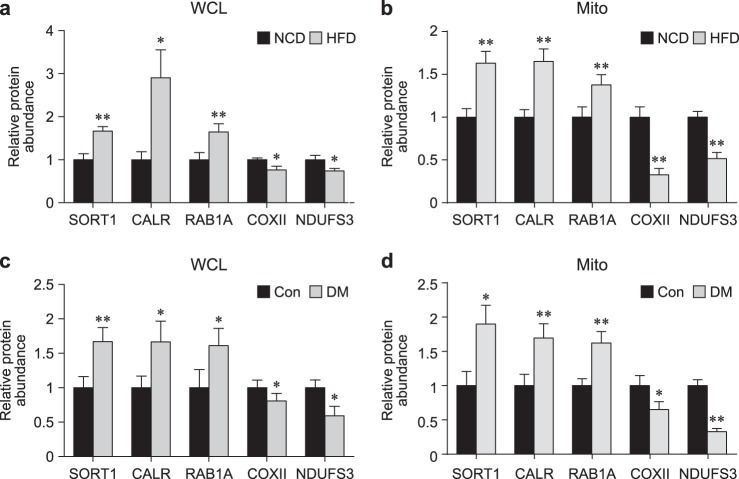
Validation of the selected proteins in independent samples. **a-d** Protein expression levels of the five selected proteins (SORT1, CARL, RAB1A, COX2, and NDUFS3) in T2DM and nondiabetic samples. SORT1, CARL, and RAB1A were upregulated in high fat diet-fed mice (diabetic conditions) compared with normal chow diet-fed mice (nondiabetic conditions), whereas COX2 and NDUFS3 were downregulated. Total proteins (**a**; WCL) and mitochondria fractions (**b**; Mito) were prepared from skeletal muscle tissues of normal chow diet- and high fat diet-fed mice (*n* = 11). Furthermore, total proteins (**c**; WCL) and mitochondria fractions (**d**; Mito) were prepared from human skeletal muscle tissues of T2DM patients (*n* = 14) and nondiabetic controls (*n* = 12). The data are shown as the mean ± SEM. *P < 0.05, **P < 0.01 by the Mann–Whitney test

To experimentally test the validity of the five selected proteins, we first confirmed the differential expression of the five selected proteins in diabetic mouse high fat diet (HFD)-induced obesity models using western blot analysis (see Materials and methods). We have previously shown that body weight, fat mass, blood glucose levels, and blood insulin levels in HFD-fed (6 weeks) mice are significantly higher than those in normal chow diet-fed mice^59^. Consistent with the LC-MS/MS data, SORT1, CALR, and RAB1A were significantly (*P* < 0.05) upregulated in both skeletal muscle tissues of HFD-fed mice (Fig. 4a; Supplementary Figure S7a) and mitochondria isolated from the skeletal muscle tissues (Fig. 4b; Supplementary Figure S7b) compared with the corresponding samples from normal chow diet-fed mice. We also confirmed that COXII and NDUFS3 were significantly (*P* < 0.05) downregulated in both skeletal muscles (Fig. 4a; Supplementary Figure S8a) and mitochondria from skeletal muscles of HFD-fed mice (Fig. 4b; Supplementary Figure S8b), consistent with the LC-MS/MS data. Moreover, to confirm the differential expression of the five selected proteins in human skeletal muscle tissues, we further collected skeletal muscle tissues from an independent set of 14 T2DM patients and 12 nondiabetic controls based on the same criteria employed for the collection of samples during the discovery phase using LC-MS/MS analysis (Supplementary Table S1; see Materials and methods). Consistent with LC-MS/MS data, SORT1, CALR, and RAB1A showed significantly (*P* < 0.05) upregulation patterns in both skeletal muscle tissues of T2DM patients (Fig. 4c) and mitochondria isolated from them (Fig. 4d) compared with the nondiabetic controls. Similarly, COX2 and NDUFS3 showed significantly (*P* < 0.05) downregulation patterns in both skeletal muscle tissues of T2DM patients (Fig. 4c) and their mitochondria (Fig. 4d) compared with the nondiabetic controls. Taken together, these results suggest that the selected mitochondrial proteome profile can serve as an indicator of T2DM-associated dysregulation of mitochondrial functions, upregulated MAM functions and downregulated OXPHOS.

## Discussion

In skeletal muscles, mitochondrial functions are essential in the pathogenesis of T2DM. Thus, a mitochondrial proteome profile indicative of mitochondrial functions can serve as a key dimension of molecular signatures to understand the pathogenesis of T2DM. Despite its potential importance, the mitochondrial proteome profile has not been systematically explored. In this study, we thus examined the comprehensive proteome profile of mitochondria isolated from skeletal muscles in T2DM patients to identify a profile representing key mitochondrial functions associated with the pathogenesis of T2DM. To achieve this goal, we developed an approach that involves (1) comprehensive label-free proteome profiling of mitochondria after the isolation of mitochondria from skeletal muscles, followed by FASP digestion and ultra-high pressure nano-LC-MS/MS analysis; (2) generation of a master AMT DB by performing offgel-based extensive fractionation; (3) identification of mitochondrial DEPs between T2DM and nondiabetic controls; (4) selection of a protein profile that can represent T2DM-associated mitochondrial functions based on functional and network analyses of the DEPs; and (5) validation of the selected protein profile in independent human and mouse T2DM samples using western blotting. Using this approach, we identified a protein profile composed of five mitochondrial proteins (three upregulated SORT1, CALR, and RAB1A representing calcium and protein transports, and two downregulated COX2 and NDUS3 representing OXPHOS).

In our mitochondrial proteome, the fraction of proteins annotated with mitochondrion, according to GOCC, was found to be 39.6% (455 of 1150 proteins; Supplementary Table 4). This fraction might be considered to be too low, thus casting doubt on our experimental protocol for the isolation of mitochondria from skeletal muscle tissues. To examine this issue, we compared the fraction in our study with those in the two previous studies. Paglianini et al.^42^ detected 3881 potential mitochondrial proteins by LC-MS/MS analyses of pure and crude fractions of mitochondria and then selected 2859 of 3881 mitochondrial proteins by removing 1022 that were relatively enriched in crude fractions of mitochondria, compared with the pure fractions. The 2859 proteins were mapped to 2464 protein-coding genes. Of the 2464 genes, 629 (25.6%) were found to be localized in mitochondria based on GOCCs. Furthermore, Lefort et al.^43^ identified 823 mitochondrial proteins that were mapped to 803 protein-coding genes. Of the 803 genes, 405 (50.4%) were found to be localized in mitochondria based on GOCCs. Thus, the average of the fractions in the two previous studies is 38.0%, which is close to the fraction (39.6%) in our study. In addition to GOCCs, the protein atlas database provides protein subcellular localization data obtained via immunofluorescence (IF) staining analysis^60^. According to these data, of our 1150 mitochondrial proteins, 277 (24.1%) were localized in mitochondria (Supplementary Table 4), and 503 (20.4%) of 2464 proteins and 316 (39.4%) of 803 proteins in the two previous studies were localized in mitochondria. As in the case of GOCCs, the fraction (24.1%) in our data was close to the average fraction (29.9%) in the two previous studies. These data suggest that our experimental protocol used for the isolation of mitochondria was as effective as those in the previous studies for identifying mitochondrial proteins using LC-MS/MS analysis.

Interestingly, large portions of 1150 detected proteins and 335 DEPs not annotated with mitochondria, based on the IF or GOCC data, were found to be localized in MAMs, according to the two MAM proteomes previously reported (Supplementary Tables 4 and 6)^61,62^. Among the 1150 detected proteins, 682 were not annotated with mitochondria according to the IF or GOCC data, and 420 (61.6%) were found to be localized to the MAMs (Supplementary Figure S9a). Furthermore, among the 335 DEPs, 184 were not annotated with mitochondria, and 109 (59.2%) were localized to the MAMs (Supplementary Figure S9b). Moreover, these 109 MAM-localized DEPs showed systematic differential expression between T2DM and NGT mitochondrial samples. These data suggest that the MAM-localized proteins, in addition to mitochondria-localized DEPs, may serve as indicators of T2DM-associated functions of mitochondria or MAMs. Thus, we included these MAM-localized DEPs when selecting the representative biomarker candidates. Among the five selected candidates, NDUFS3 and COX2 involved in OXPHOS were mitochondria-localized DEPs, while CALR, SORT, and RAB1A involved in calcium and protein transport were MAM-localized DEPs, consistent with the findings in the network models (Fig. 3d, e).

Previous studies have shown associations of the five proteins selected in this study with T2DM in insulin-secreting or responsive tissues/cells (pancreatic β cells, skeletal muscles, adipocytes, or liver tissues): (1) the amounts of SORT1 in liver and adipose tissues were associated with obesity, insulin resistance, and T2DM^63,64^, and SORT1 was found to be an essential component for the formation of GLUT4 storage vesicles in adipocytes and for the insulin-responsiveness of GLUT4 translocation to the plasma membrane;^65^ (2) the protein levels of CALR were elevated in adipose tissues of obese individuals compared with controls^66^, and the CALR gene mutation was associated with the risk of T2DM;^67^ (3) RAB1A was reported to mediate proinsulin-to-insulin conversion, and the abundance of RAB1A was decreased in pancreatic β cells of T2DM patients compared with nondiabetic controls;^68^ (4) the mRNA level of NDUFS3 was downregulated in skeletal muscles of T2DM patients compared with nondiabetic controls;^69,70^ (5) the protein level of COX was downregulated in pancreatic β cells of T2DM patients compared with nondiabetic controls and linked to insulin resistance and β cell failure^71^. However, mitochondrial associations of SORT1, CALR, and RAB1A and T2DM-associated alterations of their protein levels in the MAM or mitochondria have been never reported in insulin-secreting or responsive tissues/cells. These data indicate two aspects of the five protein panel: (1) the alterations of protein levels of these proteins in insulin-responsive tissues suggest a potential utility of the five proteins as a protein profile that can represent T2DM-associated mitochondrial functions; and (2) no associations of three upregulated proteins (SORT1, CALR, and RAB1A) with mitochondria in T2DM patients, as well as their alterations in protein levels, have previously been shown.

Several proteomic studies have described mitochondrial proteomes in various systems and/or lists of proteins localized in mitochondria. Paglianini et al.^42^ and Lefort et al.^43^ provided comprehensive mitochondrial proteomes of 14 mouse tissues and skeletal muscles, respectively (Fig. 2a). Although they could have served as useful resources for various studies of mitochondrial physiology, neither has compared their mitochondrial proteomes with those in T2DM, thus providing no lists of DEPs in T2DM compared with nondiabetic controls. Of the five selected proteins, three (RAB1A, NDUFS3, and COX2) were detected in one of these studies, but alterations of their abundances in T2DM have remained unknown. Second, several studies^8,9,47,71^ have performed comparative proteomic analyses in muscle, salivary, serum, and pancreatic islet tissues between T2DM and nondiabetic samples, providing the lists of DEPs in T2DM compared with nondiabetic controls. Of the five proteins, four (CALR, RAB1A, NDUFS3, and COX2) were detected in one of these tissues (Supplementary Table S7). Nonetheless, none of the five selected proteins has been previously reported to be altered in protein levels in mitochondria of insulin-responsive tissues.

The network analysis suggested that our mitochondrial proteome may include the proteins in MAMs (Fig. 3e, top and middle). Most of these MAM proteins were upregulated in T2DM compared with nondiabetic controls, while the mitochondrial proteins shown in Fig. 3d were largely downregulated in T2DM. These alterations in both MAM and mitochondrial proteins suggest their functional coordination in T2DM, as previously demonstrated in Alzheimer’s disease^51^. By contrast, the opposite alteration patterns (e.g., calcium and protein transport in MAM and mitochondria) may reflect possible compensatory responses for the decreased activity of the mitochondrial processes by the MAM. Interestingly, alterations of the three selected proteins (SORT1, CALR, and/or RAB1A) for the MAM processes (Fig. 3e) were reliably confirmed in skeletal muscle and mitochondrial samples from both the HFD-fed mouse model and independent human cohorts (Fig. 4a–d). Collectively, these data suggest that MAM proteins can serve as useful indicators of T2DM-associated mitochondrial functions.

Taken together, our study showed the differential expression of the five selected proteins, some of which have previously been linked to T2DM pathogenesis in other systems in mitochondria of skeletal muscle tissues from T2DM patients, thereby supporting their potential use as a protein profile that is indicative of T2DM-associated mitochondrial functions. The clinical implications of the selected proteins can be further tested with a larger number of T2DM patients. In addition, longitudinal studies can be designed to further demonstrate the nature of the dynamic changes in the proposed protein profile during the course of T2DM progression. Although we focused on the five proteins selected in this study, our approach further provided 330 other mitochondrial DEPs that may also be associated with mitochondrial functions in skeletal muscles of T2DM patients. For example, in the network models, many of the proteins involved in mitochondrial metabolic pathways (Fig. 3d) and cellular pathways in the MAM (Fig. 3e), like the five selected proteins, have been reported to be associated with T2DM-associated alterations (Supplementary Table 8). These proteins can also be used as additional indicators of T2DM-associated mitochondrial functions together with the five selected proteins. Thus, these proteins extensively extend the current list of T2DM-associated mitochondrial proteins identified using conventional small-scale experiments or approaches. This list of proteins can serve as a comprehensive resource for the study of functional links of mitochondrial functions to the pathogenesis of T2DM. Furthermore, the network models further suggest functional links of calcium and protein transport in the MAM to mitochondrial dysregulation in T2DM skeletal muscles. This understanding further suggests a potential therapeutic option involving the modulation of mitochondrial functions during the pathogenesis of T2DM. In summary, our approach effectively identified a protein profile that can provide a novel dimension of information indicative of T2DM-associated mitochondrial functions.

## Electronic supplementary material

Supplementary Information

Supplementary Table S3

Supplementary Table S4

Supplementary Table S5

Supplementary Table S6

Supplementary Table S7
