# Supplementary material for: A mitochondrial proteome profile indicative of type 2 diabetes mellitus in skeletal muscles
Source: Exp Mol Med. 2018 Sep 28;50(9):129. doi: 10.1038/s12276-018-0154-6 (PMC6162255; doi:10.1038/s12276-018-0154-6)
Supplement: Supplementary file 1 — Supplementary Information [file 12276_2018_154_MOESM1_ESM.pdf]

## Supplementary Information

## Supplementary Figures

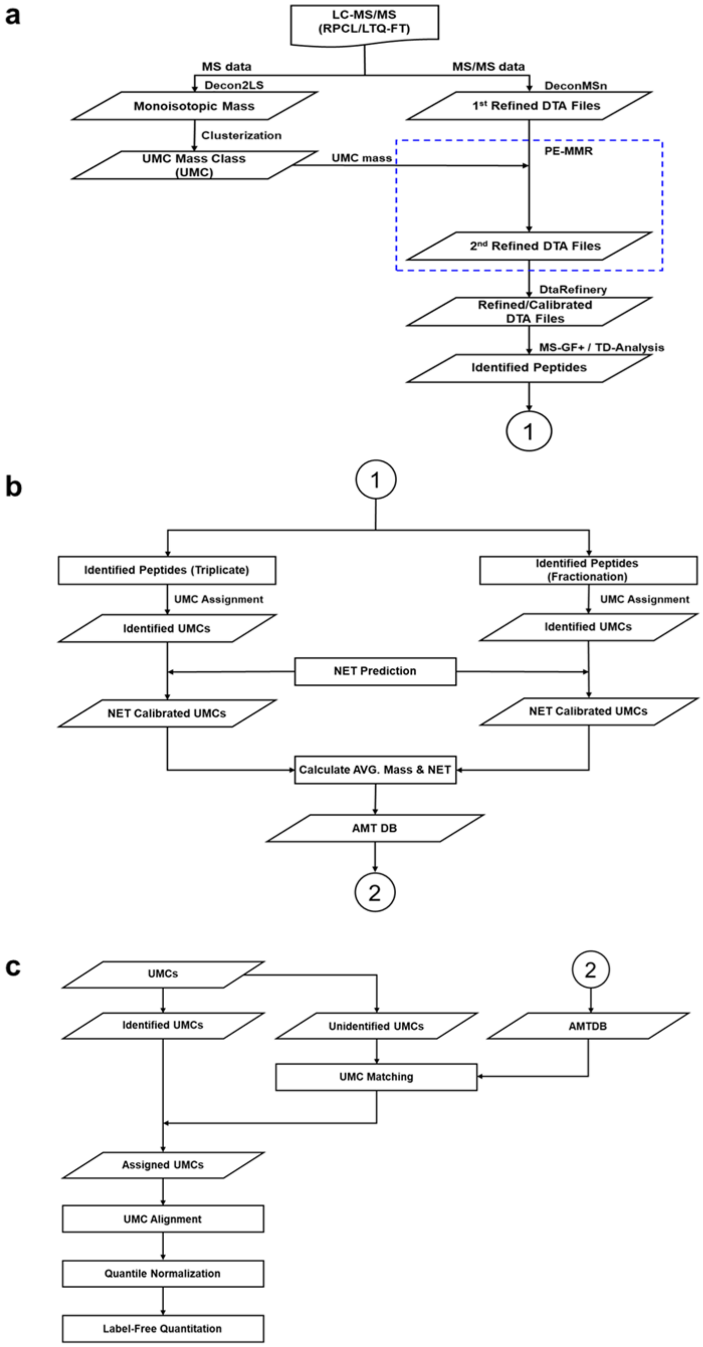


**Supplementary Figure S1. Overview of data processing. (a)** A flow chart describing the procedure for peptide identification. **(b)** A flow chart describing the procedure for accurate mass and time tag database (AMT DB) generation using the identified peptides. **(c)** A flow chart describing the procedure for peptide assignment to unidentified UMCs and alignment of the identified peptides across samples for quantitation.

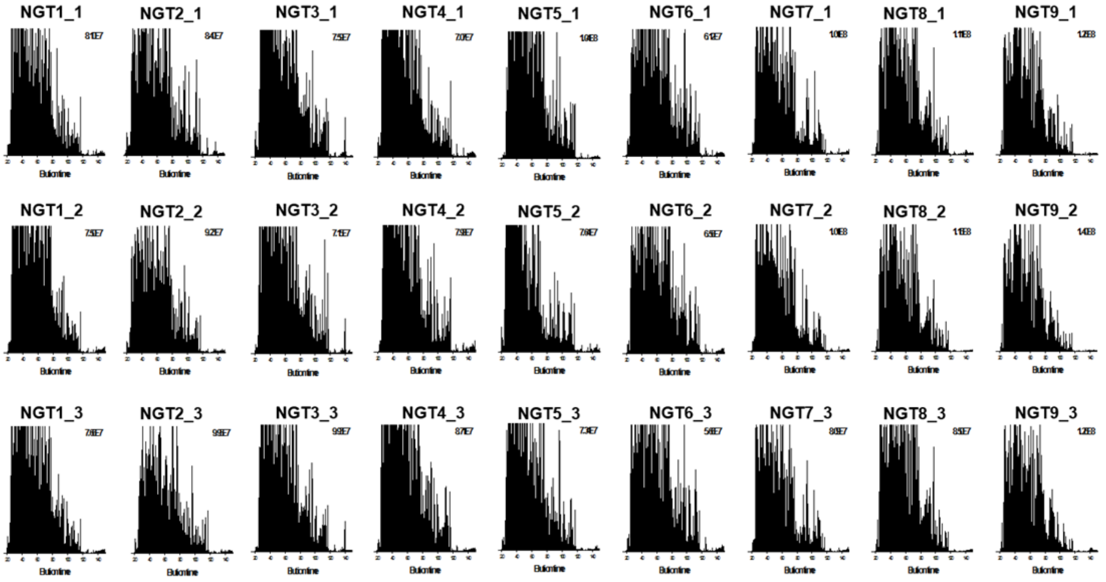


**Supplemental Figure S2. Base peak chromatograms of triplicate LC-MS/MS experiments of mitochondrial samples from nine subjects with NGT.** The nine normal samples were labeled by NGT l-9, respectively. The three technical replicates were denoted by the underscored numbers.

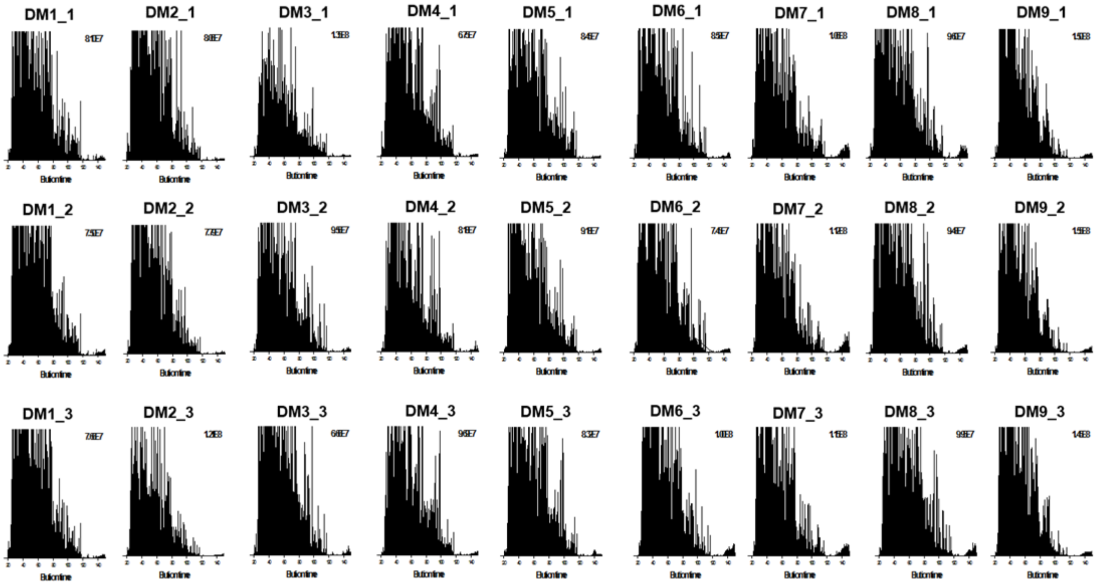


**Supplemental Figure S3. Base peak chromatograms of triplicate LC-MS/MS experiments of mitochondrial samples from nine T2DM patients.** The nine T2DM samples were labeled by DM l-9, respectively. The three technical replicates were denoted by the underscored numbers.

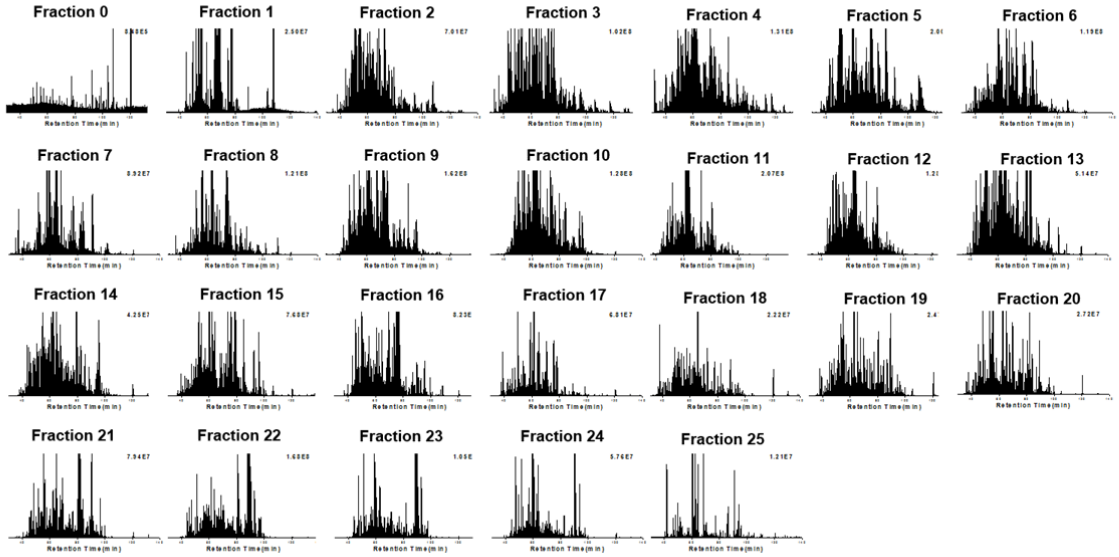


**Supplemental Figure S4. Base peak chromatograms of 26 OFFGEL fractions of pooled mitochondrial samples from the T2DM-related cybrid cells.** The 26 fractions were labeled by OG0-25, respectively.

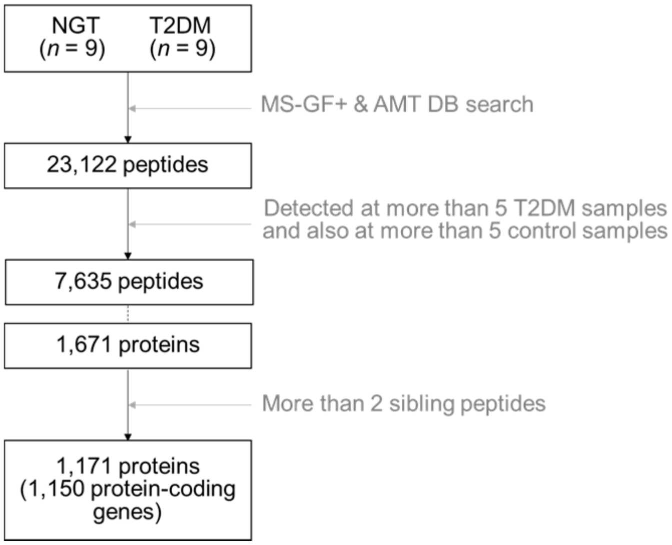


**Supplemental Figure S5. Identification of 1,171 mitochondrial proteins with high confidence.**

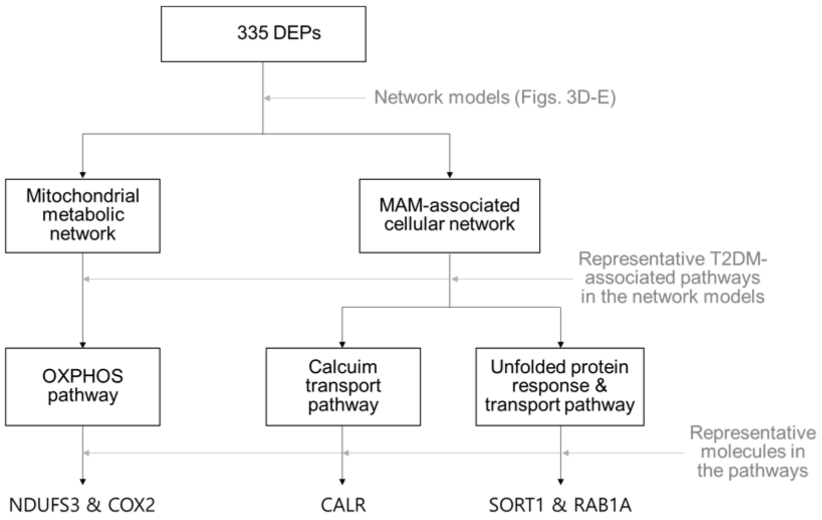


**Supplemental Figure S6. Selection of a mitochondrial protein profile indicative of T2DM-associated mitochondrial functions from network models.**

**a**

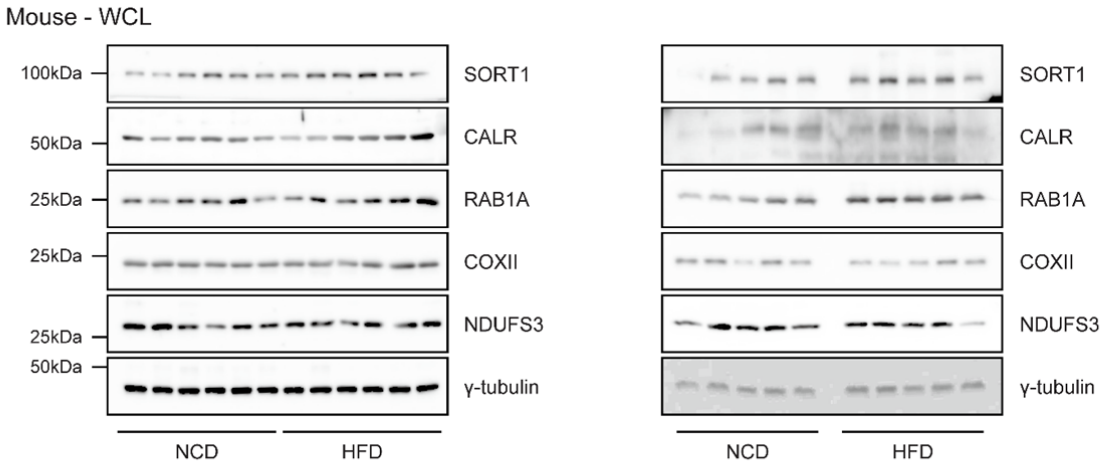


**b**

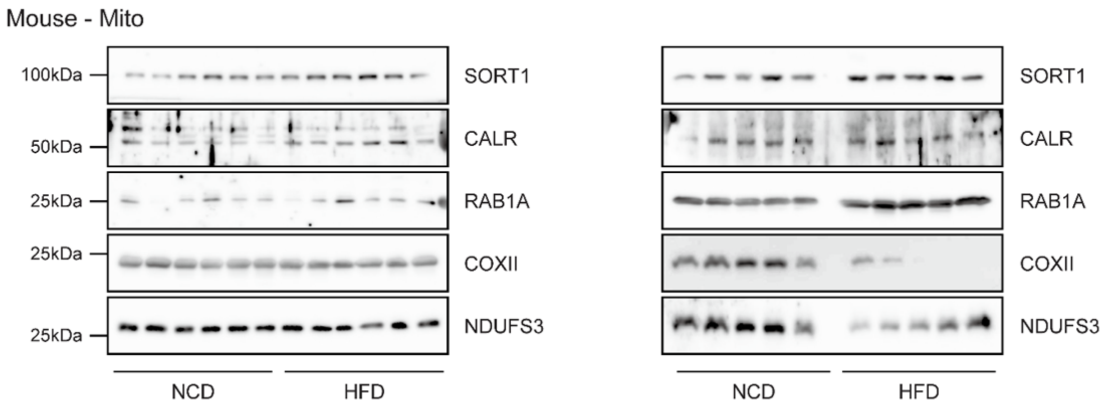


**Supplementary Figure S7. Validation of the differential expression of the selected proteins in high fat diet-fed mice (diabetic conditions) and normal chow diet-fed mice (nondiabetic conditions) using western blotting analysis.** Total proteins (**a**; WCL) and mitochondria fraction (**b**; Mito) were prepared from skeletal muscle tissues of normal chow diet- and high fat diet-fed mice (n=11).

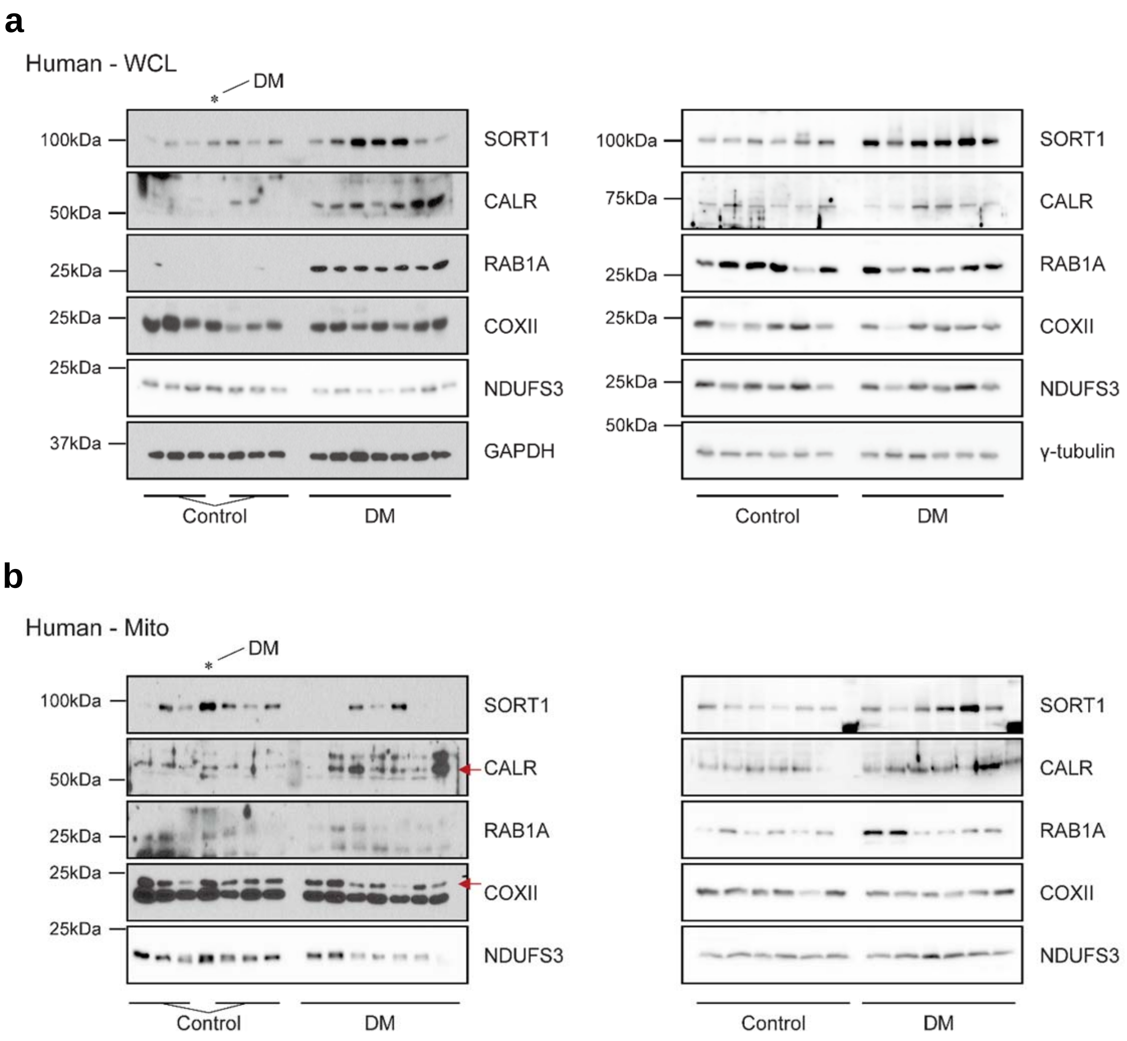


**Supplementary Figure S8. Validation of the differential expression of the selected proteins in T2DM and nondiabetic samples.** Total proteins (**a**; WCL) and mitochondria fraction (**b**; Mito) were prepared from human skeletal muscle tissues of T2DM patients (n=14) and nondiabetic controls (n=12). Note that one DM sample in the control panel was found to be diabetic at the later stage of the study.

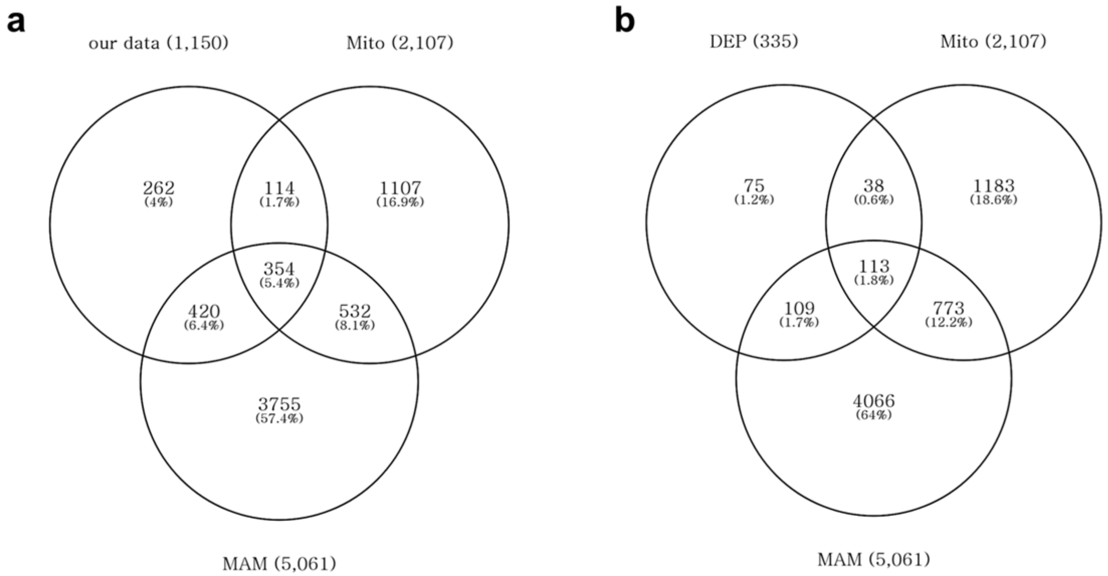


**Supplementary Figure S9. Overlaps of 1,150 detected proteins (a) and DEPs (b) with mitochondrial (Mito) or mitochondria-associated ER membrane (MAM) proteomes.** Mitochondrial proteomes include the proteins annotated with mitochondria based on subcellular localization data obtained by immunofluorescence (IF) staining analysis in the proteinatlas database or gene ontology cellular components (GOCCs). MAM proteomes include the proteins detected from two MAM proteome analyses.[1, 2]

**Supplementary Tables**

**Supplementary Table S1. Clinical characteristics of enrolled subjects in this study. (a)** Characteristics of enrolled subjects with NGT (n=9) and patients with T2DM (n=9) for initial proteome profiling. Data: mean value ± SD; * P <0.05. **(b)** Characteristics of enrolled subjects with NGT (n=12) and patients with T2DM (n=14) in the independent test set, *; P <0.05.

**Supplementary Table S1a.**

| | NGT (n=9) | DM (n=9) | p-value |
|---|---|---|---|
| Age | 56.4 ± 14.7 | 61.0 ± 14.8 | 0.52 |
| Sex (M:F) | 5:4 | 4:5 | |
| Duration of diabetes | | 8.4 ± 11.8 | |
| Body mass index (kg/m$^2$) | 24.7 ± 4.0 | 27.1 ± 3.7 | 0.20 |
| Systolic blood pressure (mm/Hg) | 129.2 ± 16.8 | 136.3 ± 15.1 | 0.36 |
| Diastolic blood pressure (mm/Hg) | 75.3 ± 13.0 | 77.6 ± 11.2 | 0.70 |
| Fasting Blood Glucose (mg/dl) | 93.9 ± 7.7 | 140.6 ± 46.3 | <0.01* |
| Hemoglobin A1c (%) | 5.5 ± 0.3 | 7.3 ± 1.3 | <0.001* |
| Total cholesterol (mg/dl) | 177.1 ± 16.8 | 223.1 ± 68.6 | 0.07 |
| BUN | 14.8 ± 5.4 | 16.2 ± 6.8 | 0.62 |
| Cr | 1.1 ± 0.4 | 1.0 ± 0.1 | 0.41 |

**Supplementary Table S1b.**

| | NGT (n=12) | DM (n=14) | p-value |
|---|---|---|---|
| Age | 56.1 ± 16.5 | 70.0 ± 12.8 | 0.03* |
| Sex (M:F) | 6:6 | 3:11 | |
| Body mass index (kg/m$^2$) | 26.1 ± 2.4 | 25.0 ± 3.3 | 0.36 |
| Systolic blood pressure (mm/Hg) | 129.8 ± 9.7 | 127.2 ± 17.6 | 0.95 |
| Diastolic blood pressure (mm/Hg) | 74.8 ± 8.3 | 70.6 ± 13.0 | 0.33 |
| Fasting Blood Glucose (mg/dl) | 100.1 ± 18.3 | 151.5 ± 65.5 | 0.01* |
| Hemoglobin A1c (%) | 5.6 ± 0.3 | 6.7 ± 1.4 | 0.02* |
| Total cholesterol (mg/dl) | 186.8 ± 43.9 | 180.1 ± 42.1 | 0.69 |
| BUN | 14.5 ± 5.5 | 18.7 ± 6.2 | 0.08 |
| Cr | 0.8 ± 0.2 | 1.1 ± 0.2 | 0.01* |

**Supplementary Table S2. The list of primary antibodies used for Western blotting analysis for the validation of the selected mitochondrial proteins.**

| Antibody target | Isotype | clone | Company | Molecular weight | Dillution concentration |
|---|---|---|---|---|---|
| Sort1 | Rabbit IgG | polyclonal | Santa Cruz | 100kDa | 1:1000 |
| CALR | Rabbit IgG | polyclonal | CellSignaling | 75kDa | 1:1000 |
| Rab1A | Rabbit IgG | polyclonal | CellSignaling | 25~30kDa | 1:2000 |
| COX II | Mouse IgG | monoclonal | MitoScience | 25kDa | 1:5000 |
| NDUFS3 | Rabbit IgG | polyclonal | Santa Cruz | 25kDa | 1:5000 |
| Tom20 | Rabbit IgG | polyclonal | CellSignaling | 15kDa | 1:5000 |
| ɣ-tubulin | Mouse IgG | monoclonal | Sigma | 45kDa | 1:10000 |

**Supplementary Tables S3-7. See the attached excel file.**

**Supplementary Table S3. The list of the 23,122 peptides identified with PSM-level FDR < 1%.** For each peptide, sequence, precursor m/z, charge state (CS), and score [-log (SpecEValue)] are shown. SpecEValue is the score obtained from MS-GF+ search engine, which represents the reliability of the PSM.

**Supplementary Table S4. 1,150 proteins identified from mitochondrial samples.** For each protein, the UniProt accession ID, EntrezID, and the number of identified unique peptides are shown. Also, whether each protein is localized in mitochondria or MAM is shown, according to both IF-based subcellular localization data and GOCCs or MAM proteomes previous reported.[1, 2]

**Supplementary Table S5. GO enrichment analysis of the 1,150 mitochondrial proteins. (A)** GOCCs and **(B)** GOBPs represented by the 1,150 mitochondrial proteins. The 'Count' represents the number of proteins with the corresponding GOBPs or GOCCs. The P-values imply the significance of being enriched by the mitochondrial proteins. The percentage represents the portion of the genes encoding the proteins with the corresponding GOBPs or GOCCs in the 1,150 mitochondrial proteins.

**Supplementary Table S6. 523 peptides that showed altered peptide abundances in T2DM compared to NGT.** UniProt IDs, Entrez IDs, symbols, and descriptions of the DEPs are shown. The ones in 'up-regulated DEPs' (or 'down-regulated DEPs') column imply that the corresponding proteins are up-regulated (or down-regulated) in T2DM, compared to normal controls. FDR values were computed as described in Methods section. Also, whether each protein is localized in mitochondria or MAM is shown, according to both IF-based subcellular localization data and GOCCs or MAM proteomes previous reported.[1, 2]

**Supplementary Table S7. GOBPs represented by in the 135 up-regulated proteins (A), and the 200 down-regulated proteins (B).** The 'Count' represents the number of proteins with the corresponding GOBPs. The P-values imply the significance of being enriched by the up- or down-regulated proteins. The percentage represents the portion of the genes encoding the proteins with the corresponding GOBPs in the up- or down-regulated proteins.

**Supplementary Table S8. List of known MAM-localized proteins.** The "O" symbol indicates that the corresponding MAM protein was detected in our data or identified as a DEP.

| Symbol | Reference | Detected in our data | DEP |
|---|---|---|---|
| ACSL1 | 3 | O | O |
| ACSL4 | 3 | O | |
| AMFR | 4 | | |
| APOB | 5 | O | |
| APOC1 | 5 | | |
| APOE | 5 | | |
| ATP2A1 | 6 | O | O |
| ATP2A2 | 6, 7 | O | O |
| ATP2A3 | 6 | O | O |
| CANX | 8 | O | |
| DGAT2 | 9, 10 | | |
| DNM1L | 11 | | |
| ERO1L | 12, 13 | | |
| ERP44 | 14 | | |
| HSPA5 | 13 | O | |
| HSPA9 | 15 | O | |
| ITPR1 | 15, 16 | | |
| ITPR2 | 17 | | |
| ITPR3 | 18, 19 | | |
| MAVS | 20 | O | |
| MFN2 | 21 | O | O |
| MTTP | 9, 22 | | |
| PACS2 | 23 | | |
| PDIA3 | 24 | O | O |
| PEMT | 25 | | |
| PML | 19 | | |
| PSEN2 | 26 | | |
| PTDSS1 | 27 | | |
| PTDSS2 | 27 | | |
| RAB32 | 28 | | |
| RYR1 | 29 | O | O |
| SCD | 22 | | |
| SEC61A1 | 20 | O | |
| SIGMAR1 | 16 | | |
| SOAT1 | 9 | | |
| TRAP | 20 | | |
| VDAC1 | 15 | O | O |

**Supplementary Table S9. Associations of the five proteins with T2DM previously reported.** The "O" symbol indicates that the corresponding protein was detected in the indicated studies.

| Selected proteins | Previous literatures | | | Global proteomic profiling | | | | | | | | | |
|---|---|---|---|---|---|---|---|---|---|---|---|---|---|
| | | | | T2DM Dataset | | | | | | | | Mitochondria Dataset | |
| | | | | Hwang et al., Diabetes (2010) | | Rao et al., JPR (2008) | | Li et al., PLOS ONE (2008) | | Lu et al., MCP (2008) | | Paglianini et. al., 2010 | Lefort et. al., 2007 |
| | | | | Muscle, human | | Salivary, human | | Serum, human | | Pancreatic islet, mouse | | 14 tissues, mouse | Skeletal muscles |
| | ref | Tissue | T2DM association | Detected | DEP | Detected | DEP | Detected | DEP | Detected | DEP | Detected | Detected |
| SORT1 | Li et. al. 2015 | Liver | Sortilin 1 (Sort1) transported apoB100 to the lysosome for degradation in the liver and thus regulate plasma cholesterol and triglyceride levels. Hepatic Sort1 was down-regulated in diabetic mice, which was partially restored after the administration of the insulin sensitizer metformin. | | | | | | | | | | |
| | Bi et. al., 2013 | Liver | Hepatic Sort1 protein was markedly decreased in mouse models of type I and type II diabetes and in human individuals with obesity and liver steatosis, whereas increasing hepatic Sort1 expression reduced plasma cholesterol and triglycerides in mice | | | | | | | | | | |
| | Shi et. al. 2005 | 3T3-L1 adipocytes | Sortilin is essential and sufficient for the formation of Glut4 and acquisition of insulin responsiveness in adipose cells | | | | | | | | | | |
| CALR | Boden et al., 2008 | Adipose tissue | The protein levels of CALR was elevated in fat biopsy samples from obese individuals. | O | | O | | | | O | O | | |
| | Mahmazi et al., 2013 | pancreatic beta cells | CALR gene mutations were associated with the pathogenesis of T2DM | | | | | | | | | | |
| RAB1A | Liu et. al., 2016 | pancreatic beta | RAB1A mediated proinsulin to insulin conversion and the abundance of RAB1A was decreased in pancreatic islets form T2DM patients. | | | | | | | O | O | | O |
| NDUFS3 | Mootha et al., 2003 | skeletal muscle | The genes involved in oxidative phosphorylation were coordinately decreased in human diabetic muscle. | O | | | | | | O | | O | O |
| COX2 | Lu et al., 2008 | pancreatic beta cells | The proteins associated with mitochondrial oxidative phosphorylation (NDUFA9, UQCRH, COX2, COX4I1, COX5A, ATP6V1B2, and ATP6V1H) were down-regulated in pancreatic beta cells from T2DM mouse model. | O | | | | | | O | O | O | O |

## Supplementary Table References


1. Horner SM, Wilkins C, Badil S, Iskarpatyoti J, Gale M, Jr. Proteomic analysis of mitochondrial-associated ER membranes (MAM) during RNA virus infection reveals dynamic changes in protein and organelle trafficking. *PLoS One* 2015; **10**(3)**:** e0117963.

2. Ma JH, Shen S, Wang JJ, He Z, Poon A, Li J *et al.* Comparative Proteomic Analysis of the Mitochondria-associated ER Membrane (MAM) in a Long-term Type 2 Diabetic Rodent Model. *Sci Rep* 2017; **7**(1)**:** 2062.

3. Lewin TM, Kim JH, Granger DA, Vance JE, Coleman RA. Acyl-CoA synthetase isoforms 1, 4, and 5 are present in different subcellular membranes in rat liver and can be inhibited independently. *J Biol Chem* 2001; **276**(27)**:** 24674-9.

4. Wang HJ, Guay G, Pogan L, Sauve R, Nabi IR. Calcium regulates the association between mitochondria and a smooth subdomain of the endoplasmic reticulum. *J Cell Biol* 2000; **150**(6)**:** 1489-98.

5. Vance JE. Phospholipid synthesis in a membrane fraction associated with mitochondria. *J Biol Chem* 1990; **265**(13)**:** 7248-56.

6. Simpson PB, Mehotra S, Lange GD, Russell JT. High density distribution of endoplasmic reticulum proteins and mitochondria at specialized Ca2+ release sites in oligodendrocyte processes. *J Biol Chem* 1997; **272**(36)**:** 22654-61.

7. Lynes EM, Bui M, Yap MC, Benson MD, Schneider B, Ellgaard L *et al.* Palmitoylated TMX and calnexin target to the mitochondria-associated membrane. *EMBO J* 2012; **31**(2)**:** 457-70.

8. Myhill N, Lynes EM, Nanji JA, Blagoveshchenskaya AD, Fei H, Simmen KC *et al.* The subcellular distribution of calnexin is mediated by PACS-2. *Mol Biol Cell* 2008; **19**(7)**:** 2777-2788.

9. Rusinol AE, Cui Z, Chen MH, Vance JE. A Unique Mitochondria-Associated Membrane-Fraction from Rat-Liver Has a High-Capacity for Lipid-Synthesis and Contains Pre-Golgi Secretory Proteins Including Nascent Lipoproteins. *J Biol Chem* 1994; **269**(44)**:** 27494-27502.

10. Stone SJ, Levin MC, Zhou P, Han JY, Walther TC, Farese RV. The Endoplasmic Reticulum Enzyme DGAT2 Is Found in Mitochondria-associated Membranes and Has a Mitochondrial Targeting Signal That Promotes Its Association with Mitochondria. *J Biol Chem* 2009; **284**(8)**:** 5352-5361.

11. Burte F, Carelli V, Chinnery PF, Yu-Wai-Man P. Disturbed mitochondrial dynamics and neurodegenerative disorders. *Nat Rev Neurol* 2015; **11**(1)**:** 11-24.

12. Anelli T, Bergamelli L, Margittai E, Rimessi A, Fagioli C, Malgaroli A *et al.* Ero1 alpha Regulates Ca2+ Fluxes at the Endoplasmic Reticulum-Mitochondria Interface (MAM). *Antioxid Redox Sign* 2012; **16**(10)**:** 1077-1087.

13. Gilady SY, Bui M, Lynes EM, Benson MD, Watts R, Vance JE *et al.* Ero1 alpha requires oxidizing and normoxic conditions to localize to the mitochondria-associated membrane (MAM). *Cell Stress Chaperon* 2010; **15**(5)**:** 619-629.

14. Higo T, Hattori M, Nakamura T, Natsume T, Michikawa T, Mikoshiba K. Subtype-specific and ER

lumenal environment-dependent regulation of inositol 1,4,5-trisphosphate receptor type 1 by ERp44. *Cell* 2005; **120**(1)**:** 85-98.

15. Szabadkai G, Bianchi K, Varnai P, De Stefani D, Wieckowski MR, Cavagna D *et al.* Chaperone-mediated coupling of endoplasmic reticulum and mitochondrial Ca2+ channels. *J Cell Biol* 2006; **175**(6)**:** 901-911.

16. Hayashi T, Su TP. Sigma-1 receptor chaperones at the ER-Mitochondrion interface regulate Ca2+ signaling and cell survival. *Cell* 2007; **131**(3)**:** 596-610.

17. Simpson PB, Mehotra S, Lange GD, Russell JT. High density distribution of endoplasmic reticulum proteins and mitochondria at specialized Ca2+ release sites in oligodendrocyte processes. *J Biol Chem* 1997; **272**(36)**:** 22654-22661.

18. Csordas G, Thomas AP, Hajnoczky G. Quasi-synaptic calcium signal transmission between endoplasmic reticulum and mitochondria. *Embo J* 1999; **18**(1)**:** 96-108.

19. Giorgi C, Ito K, Lin HK, Santangelo C, Wieckowski MR, Lebiedzinska M *et al.* PML Regulates Apoptosis at Endoplasmic Reticulum by Modulating Calcium Release. *Science* 2010; **330**(6008)**:** 1247-1251.

20. West AP, Shadel GS, Ghosh S. Mitochondria in innate immune responses. *Nat Rev Immunol* 2011; **11**(6)**:** 389-402.

21. de Brito OM, Scorrano L. Mitofusin 2 tethers endoplasmic reticulum to mitochondria. *Nature* 2008; **456**(7222)**:** 605-U47.

22. Man WC, Miyazaki M, Chu K, Ntambi J. Colocalization of SCD1 and DGAT2: implying preference for endogenous monounsaturated fatty acids in triglyceride synthesis. *J Lipid Res* 2006; **47**(9)**:** 1928-1939.

23. Simmen T, Aslan JE, Blagoveshchenskaya AD, Thomas L, Wan L, Xiang Y *et al.* PACS-2 controls endoplasmic reticulum-mitochondria communication and Bid-mediated apoptosis. *Embo J* 2005; **24**(6)**:** 717-729.

24. Li Y, Camacho P. Ca2+-dependent redox modulation of SERCA 2b by ERp57. *J Cell Biol* 2004; **164**(1)**:** 35-46.

25. Cui Z, Vance JE, Chen MH, Voelker DR, Vance DE. Cloning and Expression of a Novel Phosphatidylethanolamine N-Methyltransferase - a Specific Biochemical and Cytological Marker for a Unique Membrane-Fraction in Rat-Liver. *J Biol Chem* 1993; **268**(22)**:** 16655-16663.

26. Area-Gomez E, de Groof AJC, Boldogh I, Bird TD, Gibson GE, Koehler CM *et al.* Presenilins Are Enriched in Endoplasmic Reticulum Membranes Associated with Mitochondria. *Am J Pathol* 2009; **175**(5)**:** 1810-1816.

27. Stone SJ, Vance JE. Phosphatidylserine synthase-1 and-2 are localized to mitochondria-associated membranes. *J Biol Chem* 2000; **275**(44)**:** 34534-34540.

28. Bui M, Gilady SY, Fitzsimmons REB, Benson MD, Lynes EM, Gesson K *et al.* Rab32 Modulates Apoptosis Onset and Mitochondria-associated Membrane (MAM) Properties. *J Biol Chem* 2010; **285**(41)**:**

31590-31602.

29. Hayashi T, Rizzuto R, Hajnoczky G, Su TP. MAM: more than just a housekeeper. *Trends Cell Biol* 2009; **19**(2)**:** 81-88.
